# The Role of Non-coding RNAs in Alzheimer’s Disease: From Regulated Mechanism to Therapeutic Targets and Diagnostic Biomarkers

**DOI:** 10.3389/fnagi.2021.654978

**Published:** 2021-07-02

**Authors:** Yuan Zhang, Yanfang Zhao, Xiang Ao, Wanpeng Yu, Lei Zhang, Yu Wang, Wenguang Chang

**Affiliations:** ^1^Institute for Translational Medicine, The Affiliated Hospital of Qingdao University, Qingdao University, Qingdao, China; ^2^Institute of Biomedical Research, School for Life Science, Shandong University of Technology, Zibo, China; ^3^School of Basic Medical Sciences, Qingdao University, Qingdao, China

**Keywords:** Alzheimer’s disease, miRNAs, lncRNAs, piRNAs, circRNAs

## Abstract

Alzheimer’s disease (AD) is a progressive neurodegenerative disorder. AD is characterized by the production and aggregation of beta-amyloid (Aβ) peptides, hyperphosphorylated tau proteins that form neurofibrillary tangles (NFTs), and subsequent neuroinflammation, synaptic dysfunction, autophagy and oxidative stress. Non-coding RNAs (ncRNAs) can be used as potential therapeutic targets and biomarkers due to their vital regulatory roles in multiple biological processes involved in disease development. The involvement of ncRNAs in the pathogenesis of AD has been increasingly recognized. Here, we review the ncRNAs implicated in AD and elaborate on their main regulatory pathways, which might have contributions for discovering novel therapeutic targets and drugs for AD.

## AD Pathogenesis

Alzheimer’s disease (AD) is a central nervous system degenerative disease that is mainly characterized by the progressive deterioration of memory and cognitive functions resulting in autonomy loss (Tiwari et al., 2019). Many hypotheses about AD pathogenesis have been suggested. Currently, it is believed that the accumulation of two proteins in the brain is the key to AD. One of these key proteins is beta-amyloid (Aβ), which accumulates to abnormal levels in the brains of AD patients, forms plaques, accumulates in neurons, and disrupts cell function (Gouras et al., 2015). Senile plaques are extracellular deposits of Aβ resulting from cleavage of the transmembrane amyloid precursor protein (APP). Cleavage of the APP by α-secretase generates peptide p3, a step in the anti-amyloidogenic pathway, while cleavage of APP by the β-secretase, generates fragment C99 and the soluble APPβ. Subsequently, C99 can be cleaved by γ-secretase to produce Aβ42 (Wilkins and Swerdlow, 2017). The other key protein is hyperphosphorylated tau protein, which also accumulates to abnormal levels and forms neurofibrillary tangles (NFTs) in neurons, blocking synaptic transmission (Wang J.Z. et al., 2014). Other hypotheses explaining AD pathogenesis suggest roles for neuroinflammation, synaptic dysfunction, autophagy and oxidative stress.

Alzheimer’s disease is a complex disease involving multiple interlinked signaling pathways and cell types. There is currently no cure for AD, but attempted therapies have been made to slow disease progression or attenuate its symptoms. However, currently, an effective treatment of AD still needs to be developed. In particular, the discovery and application of novel therapeutic targets for AD have attracted increasing attention, such as non-coding RNAs involved in the pathogenesis of AD.

## The Non-Coding RNAs in AD Pathogenesis

Non-coding RNAs (ncRNAs) constitute a novel, vast and diverse family of non-protein-coding transcripts that have the potential for use as therapeutic targets and biomarkers due to their vital regulatory roles in multiple biological processes in disease development. ncRNAs interact with DNA, RNA, and protein through their primary sequence and structural elements, further regulating various biological processes, such as gene transcription, RNA turnover, mRNA translation, and protein assembly (Hombach and Kretz, 2016; Idda et al., 2018).

### Regulation Mechanism of Non-coding RNA

#### The miRNA-mRNA Networks

Gene regulation networks are complex and diverse. Recent studies examining the regulation of microRNAs (miRNAs) in AD have been relatively thorough, and mechanisms of these miRNAs have been clarified. MiRNAs are short RNAs with a length of 21–24 base pairs that play vital roles in regulating gene expression at the posttranscriptional level by binding to the 3′ untranslated region (3′-UTR) of target mRNAs to suppress their stability and translation (Cannell et al., 2008). In addition, several target mRNAs contain multiple binding sites for different ncRNAs, suggesting that individual mRNAs are regulated by many ncRNAs.

#### The PIWI/piRNA-mRNA Networks

PIWI-interacting RNAs (piRNAs) are ncRNAs with 24–32 nts that interact with piwi proteins and function in a complex to regulate cellular activities through RNA silencing. Most piRNAs are distributed in mammalian germline cells. Recent studies have found piRNAs is found in the brains of multiple species. However, piRNAs are poorly conserved, even between closely related species, and are tissue specific. Therefore, relatively little knowledge is available on the potential roles of piRNAs in species and/or the brain, even in neurodegenerative diseases (Wakisaka and Imai, 2019).

**FIGURE 1:**
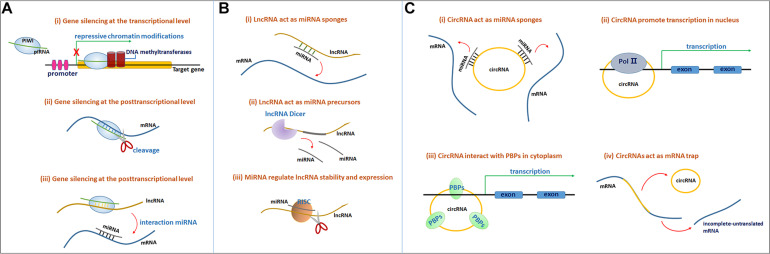
**(A)** The molecular mechanisms of the PIWI-piRNA pathway. The PIWI protein and piRNA form a complex. (i) Gene silencing at the transcriptional level. The PIWI-piRNA complex through recruiting a chromatin methylation complex to the nucleus, often serve as repressive chromatin modification. (ii) Gene silencing at the posttranscriptional level. PIWI protein serve as the endonuclease activity to inducing the cleavage of the target mRNAs in the cytoplasm. (iii) PiRNAs through binding to lncRNAs, release miRNAs from the binding sites of lncRNAs to induce target mRNA degradation. **(B)** Mechanisms of lncRNA-miRNA interaction. (i) LncRNAs act as miRNAs sponges. LncRNAs can sponges miRNAs through miRNA reaction elements (MREs) and suppress the targeting mRNAs degradation mediated by miRNAs. (ii) LncRNAs can function as miRNA precursors that generate specific miRNAs by intracellular RNA splicing and enhance the posttranscriptional regulation of target mRNAs. (iii) MiRNAs in RISCs can target and reduce some lncRNAs stability through imperfect base-pairing. **(C)** (i) CircRNAs function as miRNA sponges to bind to miRNAs and inhibit target mRNAs degradation. (ii) CircRNAs interact with the Pol II complex in the nucleus to influence mRNA transcriptional levels. (iii) CircRNAs bind to RNA-binding proteins (RBPs) to influence mRNA transcriptional levels. (iv) CircRNAs function as mRNA trap to influence mRNA splicing and protein translation.

The mechanism of PIWI-piRNA function relies on the specific sequence of the piRNA that recognizes its target genes through base-pair complementarity, silencing the target mRNA at both the transcriptional and posttranscriptional levels. The PIWI protein is an effector in the piRNA-mRNA pathway. Genes are often silenced at the transcriptional level through the induction of repressive chromatin modifications at genomic target loci and via *de novo* DNA methylation (Figure 1Ai) (Kim, 2019). Gene silencing at the posttranscriptional level often occurs through the cleavage of the target mRNA transcripts by the PIWI endonuclease (Figure 1Aii; Kim, 2019). In addition, piRNAs bind lncRNAs in a sequence-specific manner, thus releasing miRNAs from the binding sites of these lncRNAs and inducing target mRNA degradation (Figure 1Aiii; Kim, 2019).

#### The lncRNA-miRNA-mRNA Networks

Long non-coding RNAs (lncRNAs) range from 200 nts to over 100 kb, and they have been shown to play pivotal roles in numerous essential biological processes, including those leading to human diseases. LncRNAs can regulate gene expression at the epigenetic, transcriptional, and posttranscriptional level (Schmitz et al., 2016).

Long non-coding RNAs function as miRNA sponges. LncRNAs can adsorb targets in one or several miRNAs through their own miRNA reaction elements (MREs) at binding sites, further suppressing miRNA targeting of mRNAs and the degradation mediated by miRNAs (Figure 1Bi). Sponging miRNAs is among of the common posttranscriptional regulatory mechanisms of lncRNAs (Hombach and Kretz, 2016). For instance, BACE1-AS functions as a miR-761 sponge, actively suppressing the degradation mediated by miR-761 and upregulating BACE1 expression in individuals with AD (Zeng et al., 2019).

Additionally, lncRNAs function as miRNA precursors. In addition to exerting complex effects on gene expression and signal transduction, lncRNAs might also function as precursors or scaffolds for miRNAs. In this case, lncRNAs might generate specific miRNAs through intracellular RNA splicing and enhance the posttranscriptional regulation of target mRNAs (Figure 1Bii; Lekka and Hall, 2018).

Emerging studies have recently shown that miRNAs influence lncRNA function, and several lncRNAs have been shown to be regulated by miRNAs. In addition to other RNA binding protein complexes, such as the RNA-induced silencing complex (RISC), miRNAs interact with lncRNAs to regulate lncRNA stability and expression (Figure 1Biii; Lekka and Hall, 2018).

#### CircRNA-miRNA/circRNA-mRNA Networks

Recently, circular RNAs (circRNAs) have been shown to mediate several regulatory functions in various diseases. The circRNA-miRNA/circRNA-mRNA pathways play important roles in regulating the pathological process of AD (Akhter, 2018).

CircRNAs constitute a class of novel RNA molecules with a special covalent loop but no 5′ cap or 3′ tail, which are formed from precursor mRNAs by back-splicing and exon skipping (Granados-Riveron and Aquino-Jarquin, 2016). Among the four circRNAs biogenic mechanisms, intron-pairing-driven circularization is particularly important for circularization. ALU elements or flanking inverted repeats form circRNAs by intron pairing, and ecircRNAs or EIciRNAs are formed as introns are removed or retained, indicating that ALU elements play an important roles in intron-pairing-driven circularization (Zhang X.O. et al., 2014; Liu et al., 2020). However, ALU elements are absent in mice, and they usually rely on SINE elements to promote the formation of circRNAs.

CircRNAs are thought to function as endogenous sponges that efficiently bind and inhibit miRNA transcription, thereby further influencing downstream mRNA expression through posttranscriptional regulation and ultimately participating in various diseases, including AD (Figure 1Ci; Akhter, 2018; Qu et al., 2018). For example, circ_0000950 sponges miR-103 to increase the expression of PTGS2 and further increase inflammatory cytokine levels (Yang H. et al., 2019). In addition, circRNAs have consistently been shown to regulate gene expression levels. CircRNAs might interact with the Pol II complex in the nucleus or directly bind to RNA-binding proteins (RBPs) or RNA-associated proteins in the cytoplasm to form RNA-protein complexes that modulate mRNA transcription (Figure 1Cii). Furthermore, circRNAs were also confirmed to modulate gene expression trans-functionally by competing with the pre-mRNA splicing machinery. CircRNAs function as ‘mRNA traps’ to sequester the translation start site or disrupt the integrity of a mature linear RNA to produce a fragmented untranslated RNA or an inactive protein, thereby reducing the expression of the target protein (Figure 1Ciii; Qu et al., 2018).

### Non-coding RNAs in AD Pathogenesis

Identifying new targets and improving the prognosis of patients with AD require a deeper understanding of the mechanisms of this disease. To date, studies have confirmed that some ncRNAs are abnormally expressed during the pathological process of AD and subsequently regulate downstream target genes. As shown in recent studies, ncRNAs play crucial roles in the pathophysiological processes of cell proliferation and apoptosis, oxidative stress, Aβ aggregation, tau phosphorylation, neuroinflammation and autophagy, thus contributing to AD. These ncRNAs play important regulatory roles in the key signaling pathways associated with AD pathology, and strategies targeting specific ncRNAs have the potential to become a promising novel therapeutic approach for the disease (Table 1). Therefore, treatments that target ncRNAs or use ncRNA molecules might enhance the effectiveness of therapy for AD.

**TABLE 1:** Summary of ncRNAs as potential therapeutic targets for AD.

Gene	Hallmarks in AD	Expression	Source	Target gene/expression	Regulatory function	References
**MicroRNAs**						
miR-206	increase Aβ production	upregulated	hippocampus of APP/PS1 mice	BDNF ↓	Apoptosis	Tian et al., 2014
miR-613	increase Aβ production	upregulated	serum and CSF of AD patients and hippocampus of APP/PS1 mice	BDNF ↓		Li et al., 2016
miR-133b	anti-apoptosis of neurons	downregulated	serum of AD patients	EGFR		Yang Q. et al., 2019
miR-222	promote cell cycle	downregulated	cerebral cortex of APP/PS1 mice	p27^*Kip1*^ ↑	Proliferation	Wang et al., 2015
miR-98	reduce Aβ production	downregulated	hippocampus of AD mice injected scopolamine	HEY2 ↑	Oxidative stress	Chen et al., 2019
miR-330	reduce Aβ production	downregulated	brain tissue of AD mice injected D-galactose	VAV1 ↑		Zhou et al., 2018
miR-214-3p	anti-apoptosis of neurons	downregulated	hippocampus of SAMP8 AD mice and CSF of AD patients	Atg12 ↑	Autophagy	Zhang et al., 2016
miR-132/miR-212	promote Aβ production and Tau hyperphosphorylation	upregulated	lymphoblastoid cells of AD patients	SIRT1 ↓	Inflammation	Hadar et al., 2018
miR-155	induced by Aβ aggregation	upregulated	hippocampus of 3 × Tg AD mice	SOCS-1 ↓		Guedes et al., 2014; Liu D. et al., 2019
miR-339-5p	reduce Aβ production	downregulated	brains of AD cases	BACE1 ↑	Aβ production	Long et al., 2014
miR-195	reduce Aβ production	downregulated	hippocampus of SAMP8 AD mice	BACE1 ↑		Zhu et al., 2012
miR-186	reduce Aβ production	downregulated	cortex tissue of aged mice and hippocampus of AD rats injected Aβ_1__–__42_	BACE1 ↑		Kim et al., 2016
miR-16	reduce Aβ production	downregulated	brains of AD cases	BACE1 ↑		Zhong et al., 2018
miR-188-3p	reduce Aβ production	downregulated	brains of AD cases and hippocampus of *5 × FAD* TG mice	BACE1 ↑		Zhang J. et al., 2014
miR-29a/b-1	reduce Aβ production	downregulated	brains of AD cases	BACE1 ↑		Hebert et al., 2008
miR-29c	reduce Aβ production	downregulated	brains of AD cases	BACE1 ↑		Lei et al., 2015
miR-107	reduce Aβ production	downregulated	brains of AD cases and brain of APP/PS1 mice	BACE1 ↑		Jiao et al., 2016
miR-128	increase Aβ production	upregulated	cerebral cortex of 3 × Tg AD mice	PPARγ↓		Liu Y. et al., 2019
miR-144	increase Aβ production	Upregulated	brains of AD cases	ADAM10 ↓		Cheng et al., 2013
miR-140-5p	increase Aβ production	upregulated	cerebellum and hippocampus of AD cases	ADAM10 ↓/SOX2 ↓		Akhter et al., 2018
miR-384	increase Aβ production	downregulated	CSF and blood of AD patients and hippocampus of APP/PS1 AD mice	APP ↑/BACE-1 ↑	APP level	Liu et al., 2014b
miR-101	increase Aβ production	downregulated	brain of AD cases and hippocampus of mice	APP ↑		Long and Lahiri, 2011b; Barbato et al., 2020
miR-101a-3p	increase Aβ production	downregulated	brain tissue of APP/PS1 AD mice	APP ↑		Lin et al., 2019
miR-346	increase Aβ production	downregulated	brain tissue of AD cases	APP ↑		Long et al., 2019
miR-1908	promote Aβ aggregation	upregulated	plasma of AD patients	ApoE ↓	Lipid metabolism	Wang Z. et al., 2018
miR-33	increase Aβ production	upregulated	brain tissue of APP/PS1 AD mice	ABCA1 ↓		Kim et al., 2015
miR-200b/c	promote Aβ aggregation	upregulated	brain tissue of Tg2576 AD mice	S6K1 ↓	Insulin signaling	Higaki et al., 2018
miR-26b	promote Aβ aggregation	upregulated	brain tissue of APP/PS1 AD mice	IGF-1 ↓		Liu et al., 2016
miR-132/212	promote Tau hyperphosphorylation	downregulated	brain tissue of 3 × Tg AD mice	Tau ↑	Tau level	Hernandez-Rapp et al., 2016
miR-219	promote Tau hyperphosphorylation	downregulated	brains of AD cases	Tau ↑		Santa-Maria et al., 2015
miR-146a	promote Tau hyperphosphorylation	upregulated	brain tissue of AD cases and hippocampus of *5 × FAD* mice	ROCK1 ↓	Tau phosphorylation	Wang G. et al., 2016
miR-322	promote Tau hyperphosphorylation	upregulated	brain tissue of Tg2576 AD mice	BDNF ↓		Zhang et al., 2018a
miR-125b	promote Tau hyperphosphorylation	upregulated	CSF of AD patients	SphK1 ↓		Banzhaf-Strathmann et al., 2014; Ma et al., 2017
miR-512	promote Tau hyperphosphorylation	downregulated	brain tissue of AD cases	cFLIP ↑/MCL1 ↑		Mezache et al., 2015
miR-137	promote Tau hyperphosphorylation	downregulated	hippocampus and cerebral cortex of APP/PS1 AD mice	CACNA1C ↑		Jiang et al., 2018
miR-326	promote Tau hyperphosphorylation	−	brain tissue of APP/PS1 AD mice	VAV1 ↑		He et al., 2020
miR-124	synaptic deficits	upregulated	hippocampus of Tg2576 AD mice	PTPN1 ↓	Synaptic function	Wang X. et al., 2018
miR-134-5p	synaptic deficits	upregulated	hippocampus of AD rats injected Aβ_1__–__42_	CREB ↓		Baby et al., 2020
miR-10a	synaptic deficits	upregulated	hippocampus of AD rats injected Aβ_1__–__42_	BDNF ↓		Wu et al., 2018
miR-342-5p	synaptic deficits	upregulated	hippocampus of APP/PS1 AD mice	AnkG ↓		Sun et al., 2014
miR-188-5p	synaptic deficits	downregulated	brain tissues of AD cases and *5 × FAD* mice	Nrp-2 ↑		Lee et al., 2016
miR-34c	synaptic deficits	upregulated	hippocampus of SAMP8 mice and serum of AD patients	SYT1 ↓		Shi et al., 2020
**piRNAs**						
piR_38240	−	upregulated	brains of AD cases	KPNA6 ↓/ CYCs ↓	Oxidative stress	Roy et al., 2017
piR_34393	−	upregulated	brains of AD cases	CYCs ↓/ RAB11A ↓		Roy et al., 2017
**LncRNAs**						
EBF3-AS	promote apoptosis of neurons	upregulated	brain of LOAD cases and hippocampus of APP/PS1 AD mice	EBF3 ↑	Apoptosis	Magistri et al., 2015; Gu et al., 2018
NAT-Rad18	promote apoptosis of neurons	upregulated	brains tissue of AD rats injected Aβ_1__–__40_	Rad18 ↓		Parenti et al., 2007
lncRNA n336694	increase Aβ production	upregulated	brains tissue of APP/PS1 AD mice	miR-106b ↑		Huang W. et al., 2017
SOX21-AS1	increase Aβ production	upregulated	hippocampus of AD mice injected Aβ_1__–__40_	FZD3/5 ↓	Oxidative stress	Zhang et al., 2019
lncRNA-ATB	promote apoptosis of neurons	upregulated	CSF and serum of AD patients	miR-200 ↓		Wang J. et al., 2018
MEG3	reduce Aβ production	downregulated	hippocampus of AD rats injected by Aβ_25__–__35_	unknown		Yi et al., 2019
17A	increase Aβ production	upregulated	cerebral tissue of AD cases	GABABR2 ↓	Autophagy	Massone et al., 2011; Wang et al., 2019
BACE1-AS	increase Aβ production	upregulated	brains of AD cases	BACE1 ↑	Aβ production	Modarresi et al., 2011; Zhang et al., 2018c
BC200	increase Aβ production	upregulated	superior frontal gyrus of AD cases	BACE1 ↑		Mus et al., 2007
NEAT1	increase Aβ production	upregulated	brain tissues of AD mice induced by streptozocin	miR-124 ↓		Zhao et al., 2019
51A	increase Aβ production	upregulated	brains of AD cases	SORL1 ↓	APP level	Ciarlo et al., 2013
NDM29	increase Aβ production	upregulated	cerebral cortex of AD cases	unknown		Massone et al., 2012
LRP1-AS	promote Aβ aggregation	upregulated	brains of AD cases	LRP1 ↓	Aβ clearance	Yamanaka et al., 2015
**CircRNAs**						
mmu_circRNA _013636	promote Aβ aggregation	upregulated	hippocampus of SAMP8 AD mice	unknown	Oxidative stress	Huang et al., 2018b
mmu_circRNA _012180	reduce Aβ aggregation	downregulated	hippocampus of SAMP8 AD mice	unknown		Huang et al., 2018b
mmu_circRNA _017963	−	downregulated	hippocampus of SAMP8 AD mice	mmu_miR_7033-3p	Autophagosome assembly	Huang et al., 2018a
ciRS-7	increase Aβ production	downregulated	brain of AD cases	miR-7 ↑	Aβ production	Zhao et al., 2016; Shi et al., 2017
circHDAC9	increase Aβ production	downregulated	serum of AD patients and hippocampus of APP/PS1 AD mice	miR-138 ↑		Lu et al., 2019

*NS, no significant different.*

### MiRNAs in AD Pathogenesis

#### Roles of miRNAs in Proliferation and Apoptosis of Neurons

**FIGURE 2:**
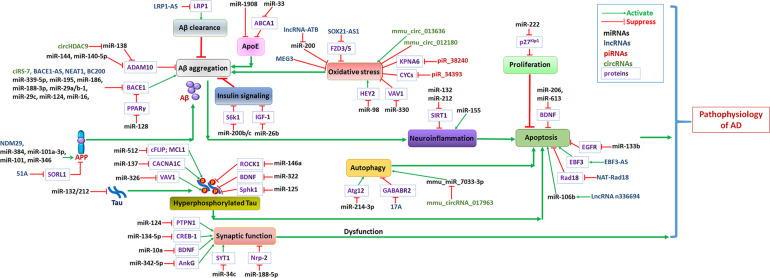
Non-coding RNA networks in AD. Abnormal levels of Aβ in the brains of AD patients formed plaques, and further induced neuroinflammation and promotes AD progression. The hyperphosphorylation of tau protein results in formation of intracellular neurofibrillary tangles induced neuron apoptosis. Oxidative stress, abnormal insulin signaling pathway or apoE functions lead to Aβ aggregation, further promoted AD progression. Growing evidence confirmed that autophagy dysfunction induce neuron apoptosis may accelerate AD progression. Green arrows represent the effects of activating downstream target genes, and red arrows represent the effects of suppressing downstream target genes.

MicroRNAs are involved in the proliferation and apoptosis of neurons by regulating downstream target genes or signaling pathways involved in the pathological process of AD (Figure 2).

Notably, miR-206 has been shown to decrease the expression of brain-derived neurotrophic factor (BDNF) by targeting the 3′-UTR of the BDNF mRNA and inhibiting its expression. BDNF is known to play a neuroprotective role against in apoptosis and to promote neuron survival, the formation of new synapses, and plasticity (Beeri and Sonnen, 2016; Lima Giacobbo et al., 2019). Tian et al. (2014) reported increased levels of miR-206 in the hippocampal tissue, cerebrospinal fluid, and plasma of APP/PS1 transgenic mice, indicating that it may contribute to neuronal apoptosis. The level of BDNF is reduced in individuals with many neurodegenerative diseases (Somkuwar et al., 2016; Rosa et al., 2019). In cultured neurons, BDNF protects against Aβ_1__–__42_ toxicity (Arancibia et al., 2008). Reduced levels of BDNF in subjects with AD have also been associated with increased pro-inflammatory cytokine activity, altered oxidative stress levels, mitochondrial dysfunction, downregulation of hippocampal neurogenesis, and GSK3β hyperactivity (Liu et al., 2015). In a study by Tian et al., miR-206 targeted the 3′-UTR of the BDNF mRNA and decreased the BDNF level in AD transgenic mouse neurons, inducing their apoptosis. Li et al. showed that miR-613 targets the 3′-UTR of the BDNF mRNA and inhibits BDNF expression, thus potentially contributing to the development of AD. The findings reported by Li et al. revealed markedly increased levels of miR-613 in the serum and cerebral spinal fluid (CSF) of patients with mild cognitive impairment (MCI) and dementia of the Alzheimer’s type (DAT), as well as in the hippocampus of APP/PS1 transgenic mice (Li et al., 2016). Moreover, the increased expression of miR-613 accompanied a significant decrease in the levels of the BDNF mRNA and protein.

According to Yang Q. et al. (2019), miR-133b might play a neuroprotective role by targeting epidermal growth factor receptor (EGFR). EGFR, a member of the ErbB family, has been reported to activate the MAPK/ERK and PI3K/AKT signaling pathways, which play crucial roles in cell survival, plasticity, and protecting neurons from neurotoxicity (Bruban et al., 2015; Liu L. et al., 2019). Recent evidence implicates this receptor in neurometabolic disorders, such as AD and aging. As shown in the study by Yang et al., the serum levels of miR-133b were substantially decreased in patients with AD and positively correlated with the MMSE score. However, increased miR-133b levels attenuated Aβ-induced neuronal apoptosis by binding to the 3′-UTR of the EGFR mRNA.

Based on accumulating evidence, some miRNAs that play important roles in cell proliferation are also associated with AD to some extent (Figure 2). Important roles for miR-222 in regulating neuron proliferation have been identified, and it might contribute to the pathogenesis and development of AD (Wang et al., 2015). Moreover, miR-222 was reported to be a direct target of p27^*Kip*1^ and was associated with cell cycle progression and proliferation (Wang et al., 2015). As shown in the study by Wang et al., miR-222 is downregulated in APPswe/PSΔE9 mice, correlating with the extent of increase in the level of the p27^*Kip*1^ protein. Importantly, p27^*Kip*1^ is a direct target of miR-222. Its expression inhibits the phosphorylation of pRb and arrests cell proliferation in G1 phase, thereby contributing to the pathogenesis of AD.

Thus, these miRNAs may be helpful for exploring new treatment strategies for AD by regulating apoptosis or cell proliferation signaling pathways.

#### Roles of miRNAs in Oxidative Stress

Oxidative stress has been recognized as a contributing factor in aging and in the progression of AD (Wang X. et al., 2014). Oxidative stress is caused by mitochondrial dysfunction and increased production of reactive oxygen species (ROS). The increase in ROS levels affects the expression and processing of AβPP, initiating Aβ accumulation and the activation of various signaling pathways in brain tissue, further contributing to the development of AD (Yuan et al., 2018; Figure 2).

The expression of miR-98 is reduced with increased levels of hairy and enhancer of split (Hes)-related with YRPW motif protein 2 (HEY2) in AD mice, and this effect is associated with the activation of oxidative stress and mitochondrial dysfunction (Chen et al., 2019). HEY2, a transcription factor for Notch, is expressed at higher levels in individuals with some diseases. Notch signaling is closely related to the differentiation, proliferation, apoptosis and oxidative stress of several cell types (Wu et al., 2016; Bai et al., 2017; Yuan et al., 2019). The expression of miR-98 suppresses the production of Aβ and ameliorates oxidative stress and mitochondrial dysfunction by targeting and inhibiting HEY2 to inactivate the Notch signaling pathway in AD mice (Chen et al., 2019).

Another miRNA that is downregulated in the neurons of AD mice is miR-330. VAV1, a target of miR-330, is upregulated in AD mice (Zhou et al., 2018). VAV1, a conserved member of the Rho family of GTPases, functions as a crucial regulator of several neurological diseases (Garcia et al., 2012; Fry et al., 2014). Upregulation of miR-330 reduces VAV1 expression and simultaneously reduces the levels of ERK1, JNK1, P38 MAPK, and Aβ. The MAPK signaling pathway plays a crucial role in the uptake and accumulation of Aβ via the α7 nicotinic acetylcholine receptor in individuals with AD (Yang et al., 2014). As shown in a study by Zhou et al., the overexpression of miR-330 contributes to reductions in the Aβ level, oxidative stress and mitochondrial dysfunction by decreasing the level of VAV1 and activation of the MAPK signaling pathway (Zhou et al., 2018).

#### The Role of miRNAs in Regulating Autophagy

The autophagy process is the major cellular degradation pathway for long-lived proteins and organelles. Autophagy in neurons is essential for the maintenance of neuronal homeostasis, and autophagy dysfunction might contribute to the development of AD (Salminen et al., 2013; Li et al., 2017). The expression and function of specific ncRNAs are closely associated with autophagy in subjects with AD (Figure 2).

For instance, miR-214-3p is a functional miRNA that inhibits autophagy and apoptosis in hippocampal neurons by targeting the 3′-UTR of the Atg12 mRNA (Zhang et al., 2016). Atg12 plays a crucial role in the early step of autophagosome formation (Tanida, 2011; Romanov et al., 2012). Zhang et al. revealed reduced miR-214-3p levels in the cerebrospinal fluid of patients with sporadic AD and in hippocampal neurons from SAMP8 mice upon the induction of autophagy. Overexpression of miR-214-3p in primary neurons increases their viability and inhibits their apoptosis and autophagy by negatively regulating Atg12 expression (Zhang et al., 2016).

#### The Role of miRNAs in Regulating Neuroinflammation

Neuroinflammation exerts important effects on cognitive functions in the pathophysiological processes of AD. In individuals with AD, the aggregation of Aβ results in the chronic activation of primed microglia, increasing the production of inflammatory cytokines and chemokines, which induces neuroinflammation (Marttinen et al., 2018; Figure 2). Based on accumulating evidence, several ncRNAs might regulate the intracellular pathways of numerous mediators of neuroinflammation.

For instance, miR-132 and miR-212 are associated with neuroinflammation in subjects with AD. Hadar et al. revealed the upregulation of miR-132 and miR-212 in lymphoblastoid cells (LCLs) from patients with AD, and correspondingly, the expression of silent information regulator 1 (sirtuin1, SIRT1) is decreased (Hadar et al., 2018). SIRT1 was previously verified to play a vital role in preventing the apoptotic death of neurons by regulating p53 and peroxisome proliferator-activated receptor-gamma coactivator 1α (PGC-1α), which decrease the accumulation of Aβ and attenuate mitochondrial dysfunction (Wang et al., 2013). Recently, mice overexpressing SIRT1 exhibited ameliorated clinical symptoms of autoimmune encephalomyelitis and attenuated axonal injury (Martin et al., 2015). SIRT1 is a target of miR-132 and miR-212, and its expression is inhibited in LCLs from patients with AD. In addition, Liu D. et al. (2019) observed increased expression of miR-155 in the hippocampus of AD rats, accompanied by increasing levels of proinflammatory cytokines. The silencing of miR-155 decreases caspase-3 levels and suppresses the inflammatory signaling pathways, ameliorating learning impairments in AD rats. Guedes et al. also identified SOCS-1 as a target of miR-155, and its expression is decreased upon miR-155 upregulation in 3 × Tg AD mice. The expression of miR-155 contributes to the production of inflammatory mediators, such as IL-6 and IFN-b, in microglia and astrocytes (Guedes et al., 2014).

#### The Role of miRNAs in Regulating the Activity of the Secretases Required for Aβ Production

Abnormalities in Aβ production and metabolism are the main causes of amyloid plaque deposition in the brain. In the brain, amyloid precursor proteins are usually subjected to posttranslational proteolytic processing by different secretases, including α-, β-, and γ-secretase. α-Secretase generates soluble amyloid protein, while β- and γ-secretases produce the β-amyloid precursor protein (βAPP), generating the amyloidogenic peptide Aβ (De Strooper et al., 2010). Dysfunction of these secretase enzymes induces abnormal Aβ deposition in the brains of patients with AD. Recently, several ncRNAs have been shown to be involved in Aβ pathology by regulating the enzymes that process Aβ.

##### The role of miRNAs in regulating BACE1

Secretases are essential participants in Aβ production. β-Secretase 1 (BACE1) catalyzes the rate-limiting, initial cleavage at the β site of APP and is followed by sequential intra-membrane processing at multiple sites by γ-secretase in the generation of Aβ (Yan and Vassar, 2014). BACE1 has been identified as a crucial enzyme in the production of the Aβ peptide during the pathophysiology of AD. Inhibition of BACE1 activity and the subsequent reduction in Aβ levels may cure or prevent AD. Numerous ncRNAs have been shown to regulate the level and function of BACE1 and subsequently influence Aβ generation (Figure 2).

The expression of crucial miRNAs that are potentially involved in regulating the expression of BACE1 appears to be decreased in the brains of patients with AD. For instance, the expression of miR-107, miR-29a/b-1, miR-29c, miR-188-3p, miR-339-5p, miR-195, miR-186, and miR-16 is decreased in the brains of patients with AD and AD mice (Hebert et al., 2008; Zhu et al., 2012; Long et al., 2014; Zhang J. et al., 2014; Lei et al., 2015; Jiao et al., 2016; Kim et al., 2016; Zhong et al., 2018). Studies have confirmed that these miRNAs regulate BACE1 expression levels by targeting the 3′-UTR region of the BACE1 mRNA. For example, miR-188-3p is capable of functionally binding to the 3′-UTR of the BACE1 mRNA, and the overexpression of miR-188-3 in the hippocampus of *5XFAD* TG mice suppresses BACE1 expression to reduce Aβ production and prevent synaptic dysfunction (Zhang J. et al., 2014). However, the downregulation of miR-188-3p expression by a miR-188-3p sponge increases BACE1 expression and induces Aβ generation and neuroinflammation. The levels of miR-29a/b-1 and miR-29c were decreased in the brains of patients with sporadic AD in other studies, and levels of both the BACE1 mRNA and protein were increased by targeting BACE1 expression through these miRNAs (Hebert et al., 2008; Lei et al., 2015). The overexpression of miR-29a/b-1 or miR-29c significantly reduces the level of the BACE1 protein and suppresses APPβ accumulation in an AD cell model (Hebert et al., 2008; Lei et al., 2015). In addition, Jiao et al. identified some drugs that are potential treatments for AD by modulating specific miRNAs to suppress BACE1 expression. Notably, miR-107 targets BACE1 and suppresses its expression, further inhibiting the production of Aβ. Osthole exerts neuroprotective effects by upregulating miR-107 and downregulating BACE1 expression *in vitro* and in AD mice (Jiao et al., 2016). In summary, these miRNAs play an important role in regulating BACE1 protein levels. Studies aiming to elucidate the complex network of regulatory miRNAs that suppress BACE1 production will lead to the discovery of effective AD therapies.

An association between miR-128 and the accumulation of Aβ was identified because this miRNA targets peroxisome proliferator-activated receptor gamma (PPARγ) in the cerebral cortex of 3 × Tg AD mice (Liu Y. et al., 2019). PPARγ regulates the transcription of BACE1 and thus inhibits the expression of the BACE1 protein, further suppressing Aβ generation (Quan et al., 2019). The level of miR-128 is increased and negatively associated with PPARγ expression in AD mice. Therefore, knockout of miR-128 may have inhibited amyloid plaque formation and Aβ generation by inactivating the APP amyloidogenic processing pathway in AD mice, leading to the upregulation of PPARγ expression (Liu Y. et al., 2019).

##### The role of miRNAs in regulating ADAM10

Alzheimer’s disease-related disintegrin and metalloproteinase 10 (ADAM10) is the major α-secretase in neurons that cleaves APP and inhibits Aβ generation. Upregulation of the level or activity of ADAM10 modulates synaptic activity and neuroprotection in subjects with AD (Yuan et al., 2017). Thus, some miRNAs have become potential targets for the treatment of AD because they regulate the level of ADAM10 (Figure 2).

For instance, Cheng et al. (2013) revealed an increased level of miR-144 in patients with AD that was associated with decreased levels of the ADAM10 protein via its interaction with the 3′-UTR of the ADAM10 mRNA. Moreover, activator protein-1 (AP-1) upregulated the level of miR-144 to suppress ADAM10 expression after Aβ treatment *in vitro* and Aβ deposition *in vivo*. In addition, Akhter et al. showed that miR-140-5p suppressed ADAM10 expression by targeting the 3′-UTR of the ADAM10 mRNA, and its expression was elevated in the hippocampus of the postmortem brain of patients with AD (Akhter et al., 2018). Furthermore, the expression of the mRNA encoding the sex-determining region Y-box 2 (SOX2) transcription factor is regulated through the interaction of its 3′-UTR with miR-140-5p. The elevated levels of miR-140-5p observed in patients with AD may also contribute to lower levels of the ADAM10 protein via SOX2. Based on these findings, a decrease in ADAM10 levels induced by specific miRNAs is postulated to induce neurotoxic Aβ. Decreases in the levels of these miRNAs may be beneficial for the prevention and treatment of AD.

#### Regulatory Effects of miRNAs on APP Levels

Numerous miRNAs have been shown to regulate the expression of the APP mRNA in human cells *in vitro*. APP is a single-pass transmembrane protein expressed in many tissues. APP is implicated in synapse formation and the plasticity of neural tissues (Turner et al., 2003; Wang S. et al., 2016). Several miRNAs have been shown to influence APP biosynthesis, thus generating Aβ plaques. These miRNAs, including miR-101, miR-101a-3p, miR-346, and miR-384, were reported to be downregulated in animal models (Figure 2). Liu et al. reported decreased expression of miR-384 along with increasing levels of APP in the CSF of patients with dementia of the Alzheimer’s type (DAT). However, overexpression of miR-384 suppresses the expression of the APP and BACE1 mRNAs and proteins by binding the 3′-UTRs of their mRNAs (Liu et al., 2014b). The binding of miR-101 and miR-101a-3p to the 3′-UTR of the APP mRNA was confirmed, which reduced the level of APP in subjects with AD (Long and Lahiri, 2011b; Lin et al., 2019; Barbato et al., 2020). Long et al. observed a significant decrease in miR-346 expression in patients with AD, particularly patients presenting with later Braak stages. Importantly, miR-346 specifically targets the 5′-UTR of the APP mRNA to increase APP translation and Aβ production (Long et al., 2019). The suppressive effects of the miRNAs on APP expression may provide a new direction for targeted therapy for AD.

#### miRNAs Associated With ApoE

MicroRNAs involved in Aβ production induced by abnormal apolipoprotein E (ApoE) expression and functions, which contributes to AD pathogenesis (Figure 2). ApoE, the major component of high-density lipoprotein (HDL)-like particles, is required for the transfer of cholesterol and phospholipids between cells (Pamir et al., 2016). ApoE is a major risk factor for AD, particularly in patients with LOAD, and the lipid metabolism regulated by ApoE in the brain might play a critical role in AD pathogenesis (Schmechel et al., 1993).

For example, miR-1908 was shown to directly interact with the 3′-UTR of the ApoE mRNA and subsequently reduced the levels of the ApoE mRNA and protein (Wang Z. et al., 2018). Wang et al. observed increased miR-1908 levels in patients with AD, and this increase was negatively correlated with the plasma ApoE level. Therefore, miR-1908 expression inhibits ApoE expression and suppresses apoE-mediated Aβ clearance.

The upregulation of miR-33 has been observed in the brains of AD mice, effectively suppressing ATP-binding cassette transporter A1 (ABCA1) expression by binding to the 3′-UTR of the ABCA1 mRNA in neural cells (Kim et al., 2015). ABCA1 is a key regulator of ApoE lipidation, which is required for the effective clearance of Aβ from neurons. Overexpression of miR-33 impairs cellular cholesterol efflux and dramatically increases extracellular Aβ levels by promoting Aβ secretion and impairing Aβ clearance from neurons. Genetic deletion of miR-33 markedly upregulates ABCA1 levels, further decreasing Aβ secretion by inhibiting the β-site cleavage of APP and promoting Aβ clearance from neurons. Thus, miR-33 may represent a potential therapeutic target for AD by regulating the metabolism of ApoE and Aβ (Kim et al., 2015).

#### miRNAs Regulate Insulin Signaling in Individuals With AD

Recently, insulin signaling was shown to be involved in regulating neural plasticity and memory processing by protecting neurons from oxidative stress and apoptosis, which were determined to play crucial roles in the brains of patients with AD (Chen et al., 2016). Deregulation of insulin, insulin receptor, insulin-like growth factor (IGF-1), and other components of the insulin signaling pathway in the brain leads to memory impairment, tau hyperphosphorylation, and Aβ accumulation (Stohr et al., 2013; Chatterjee et al., 2019; Figure 2). Moreover, the effects of miRNAs on the insulin signaling pathway and insulin resistance has recently attracted attention (Chen et al., 2016).

According to a previous study, miR-200b/c might contribute to reducing Aβ secretion and alleviating cognitive impairment by promoting insulin signaling in the AD brain (Higaki et al., 2018). The levels of both miR-200b and miR-200c are increased in primary murine neuronal cells (PMNCs) stimulated with Aβ_1__–__42_. Notably, miR-200b/c suppresses S6K1 expression at the posttranscriptional level and reduces Aβ-induced insulin resistance. S6K1 negatively regulates the PI3K/mTOR pathway by phosphorylating serine residues in insulin receptor substrate 1 (IRS1), leading to insulin resistance (Tremblay et al., 2007). AD mice overexpressing miR-200b/c showed decreased generation of Aβ and a greater ability to memorize the spatial location upon the amelioration of insulin resistance. Thus, miR-200b/c protects against Aβ-induced toxicity.

The upregulation of miR-26b has been observed in the temporal cortex of humans with AD, a condition associated with reduced levels of IGF-1 protein and increased Aβ production (Liu et al., 2016). The reduction in the level of IGF-1 associated with an accelerated accumulation of Aβ is a feature of the AD brain. IGF-1 plays an important role in the clearance of Aβ from the brain by mediating insulin/IGF-1 signaling. The astrocyte-derived IGF1 acts as a transporter, which can transport the intracellular toxic Aβ oligomers to extracellular in the form of endocytic processing (Pitt et al., 2017). The downregulation of miR-26b in neurons increases the level of the IGF-1 protein and suppressed Aβ production.

#### The Regulatory Effects of miRNAs on Tau Expression and Tau Phosphorylation

Tau is a microtubule-associated protein that is normally located in neuronal axons and associated with the cytoskeleton and the stabilization of microtubules. The abnormal expression and aggregation of hyperphosphorylated tau leads to the formation of neurofibrillary tangles (NFTs), which are a common pathological feature of AD (Li et al., 2007).

Several studies have described the direct regulation of tau expression by miRNAs (Figure 2). Smith et al. revealed decreased expression of the miRNA cluster miR-132/212 in individuals with AD (Hernandez-Rapp et al., 2016). Their subsequent study identified tau as a direct target of miR-132, and a miR-132/212 deficiency in mice led to increased tau expression, phosphorylation and aggregation. However, the overexpression of miR-132 in 3 × Tg AD mice induces a significant reduction in tau phosphorylation and attenuates long-term memory deficits. Another study reported a decrease in miR-219-related regulation of tau expression in subjects with AD. Importantly, miR-219 directly targets the 3′-UTR of tau to alter the expression of the tau mRNA at the posttranscriptional level (Santa-Maria et al., 2015).

Other miRNAs have been reported to play roles in modulating tau phosphorylation and subsequent tangle formation (Figure 2). For example, miR-146a, miR-322, and miR-125b are upregulated, and miR-512 and miR-137 are downregulated in the brains of patients with AD and mouse models, changes that are associated with the tau phosphorylation status. According to Wang et al., the increased expression of miR-146a might contribute to abnormal tau hyperphosphorylation in the brains of patients with AD (Wang G. et al., 2016). The rho-associated coiled-coil-containing protein kinase 1 (ROCK1) gene is a target of miR-146a in neural cells, and overexpression of miR-146a induces tau hyperphosphorylation by inhibiting ROCK1. ROCK1 has been shown to promote tau dephosphorylation by binding and phosphorylating the phosphatase and tensin homolog (PTEN) protein (Hu et al., 2019). Thus, the inhibition of miR-146a in a mouse model of AD reduces tau hyperphosphorylation and enhances memory function by regulating the ROCK1-PTEN signaling pathway (Wang G. et al., 2016). Recently, miR-322 expression was shown to be significantly increased, along with a decrease in BDNF expression in the AD mouse brain. BDNF plays a crucial role in regulating synaptic plasticity and neuronal survival and was reported to be significantly decreased in AD brains. BDNF-TrkB receptor activation is negatively regulated by miR-322 because it targets BDNF, thereby promoting tau phosphorylation (Zhang et al., 2018a). A significant increase in miR-125b expression was reported in patients with AD compared to healthy controls and was associated with the induction of tau phosphorylation (Banzhaf-Strathmann et al., 2014; Ma et al., 2017). The overexpression of miR-125b causes tau hyperphosphorylation by increasing the levels of P35, Cdk5, and p44/42-MAPK (Banzhaf-Strathmann et al., 2014). In addition, miR-125b may regulate AD and neuron growth and apoptosis by modulating the levels of inflammatory factors and oxidative stress through sphingosine kinase 1 (SphK1) (Jin et al., 2018). SphK1 is a key enzyme that regulates ceramide/sphingosine-1-phosphate (S1P), which mediates reactive oxygen species (ROS) production and plays a protective role in neurodegenerative diseases (Lin et al., 2016; Motyl and Strosznajder, 2018). As shown in the study by Jin et al., miR-125b directly targets SphK1 to suppress the level of the SphK1 protein, enhance oxidative stress, and increase levels of the amyloid precursor protein (APP), β-site APP cleaving enzyme 1 (BACE1) and tau proteins (Jin et al., 2018). Therefore, if SphK1 is overexpressed upon miR-125b silencing, learning and memory are enhanced and Aβ deposition is reduced in subjects with AD.

In addition, the levels of several miRNAs, including miR-512 and miR-137, are significantly reduced in the AD brain and accompanied by an abundance of hyperphosphorylated tau (Figure 2). As shown in a study by Mezache et al., the reduction in miR-512 expression and the increase in the expression of 2 of its antiapoptosis-related targets, cFLIP and MCL1, are associated with tau hyperphosphorylation in AD brains (Mezache et al., 2015). Jiang et al. revealed reduced expression of miR-137 along with increased expression of the calcium voltage-gated channel subunit alpha-1 C (CACNA1C) protein in the hippocampus and cerebral cortex of AD mice. Notably, miR-137 inhibited the phosphorylation of tau (p-tau) induced by Aβ_42_ in SH-SY5Y cells by directly binding to the 3′-UTR of the CACNA1C mRNA and suppressing the expression of its protein (Jiang et al., 2018). Moreover, miR-326 also inhibits tau phosphorylation and enhances the cognitive function of mice with AD. The expression of miR-326 negatively regulates the expression of VAV1 by binding to the 3′-UTR of the VAV1 mRNA and inhibiting the activation of the JNK signaling pathway to enhance cell viability and suppress neuronal apoptosis (He et al., 2020).

#### Regulatory Effects of miRNAs on Synaptic Function

Synaptic loss-induced memory impairments are an early pathological feature of AD and are thought to be induced by the accumulation of Aβ peptides. Aβ weakens synaptic transmission and disrupts long-term potentiation (LTP) (Mucke and Selkoe, 2012; Tanaka et al., 2019). Roles for miRNAs in controlling the expression of genes related to synaptic transmission, plasticity, and cognition, which are involved in the pathology of AD, have been reported (Figure 2).

Wang X. et al. (2018) reported a marked increase in the level of miR-124 in the temporal cortex and hippocampus of patients with AD and in the hippocampus of Tg2576 mice. The dramatic reduction in levels of the tyrosine protein phosphatase non-receptor type 1 (PTPN1) protein in the AD brain and a significant negative correlation between PTPN1 and miR-124 were observed in the temporal cortex. Wang et al. also found that miR-124 directly targets the 3′-UTR of the PTPN1 mRNA, an important tyrosine phosphatase, to suppress its translation, thereby impairing the function of AMPA-type glutamate receptors (AMPARs) and disrupting synaptic transmission, plasticity, and memory. The overexpression of miR-124 suppresses the expression of PTPN1, which dramatically induces synaptic impairments and memory deficits.

Baby et al. (2020) described the association of miR-134-5p expression with impaired synaptic plasticity in individuals with AD. The level of miR-134-5p was dramatically increased in the Aβ_1__–__42_-treated hippocampus. In this study, miR-134-5p directly targeted cAMP-response-element binding protein (CREB-1) and posttranscriptionally downregulated the expression of CREB-1 and BDNF in individuals with AD (Baby et al., 2020). CREB-1 and BDNF are two of the most important plasticity-related proteins (PRPs) that mediate functional and structural plasticity (Caracciolo et al., 2018). BDNF transcription is activated by the phosphorylation of CREB following Ca^2+^ influx in postsynaptic neurons and results in CREB binding to CRE in the BDNF gene (Tao et al., 1998; Esvald et al., 2020). Knockdown of miR-134-5p increases the levels of the CREB-1 protein and CREB phosphorylation, eventually resulting in an increase in BDNF gene transcription and the rescue of the Aβ_1__–__42_-induced impairment in synaptic function in the hippocampus of AD rats (Baby et al., 2020).

Another miRNA that directly targets BDNF is miR-10a, and it acts as a mediator of synaptic plasticity by inhibiting the BDNF-TrkB signaling pathway. Wu et al. (2018) revealed increased miR-10a levels and decreased BDNF, CREB, p38, PSD95, and TrκB expression in AD rats.

Sun et al. documented the upregulation of miR-342-5p in APP/PS1 mice that mechanistically leads to impaired synaptic function. Overexpression of miR-342-5p downregulates the expression of ankyrin G (AnkG), a protein that is known to play a critical role in the axon initial segment (Sun et al., 2014). Direct binding of miR-342-5p to the 3′-UTR of the AnkG mRNA decreases AnkG levels through repressed translation in neurons from AD transgenic mice.

Lee et al. (2016) identified a role for miR-188-5p in promoting the neurogenesis and synaptic functions of neurons, and its expression is reduced in brain tissues from patients with AD and 5XFAD mice. Furthermore, neuropilin-2 (Nrp-2), a target of miR-188-5p, is upregulated in brain tissues from patients with AD and 5XFAD mice. Nrp-2, together with its ligand semaphorin-3F, was previously reported to function as a negative regulator of spine development and synaptic structure (Tran et al., 2009). However, the expression of miR-188-5p reversed the Aβ-mediated reduction in dendritic spine density and basal synaptic transmission in 5XFAD mice by targeting the 3′-UTR of the Nrp-2 mRNA and subsequently reducing the level of the Nrp-2 protein.

Shi et al. observed a significant increase in miR-34c levels in the serum of patients with MCI and in the hippocampus of SAMP8 mice; this ncRNA plays critical roles in decreasing synaptic function in individuals with AD. Increased miR-34c expression was mediated by the ROS-JNK-p53 pathway and negatively regulated synaptotagmin 1 (SYT1) expression by targeting the 3′-UTR of the Syt1 mRNA in subjects with AD, and inhibition of miR-34c improves the memory deficits in an AD model (Shi et al., 2020).

Taken together with the aforementioned studies, the dysregulation of specific miRNAs contributes to the pathogenesis of AD by inducing synaptic dysfunction and cognitive deficits.

### piRNAs in AD Pathogenesis

#### Roles of piRNAs in Oxidative Stress

Recently, specific dysregulated piRNAs have been reported to be associated with the function of AD-related biological pathways, playing important roles in apoptosis and oxidative stress and in regulating Aβ levels in individuals with AD (Figure 2). Roy et al. observed increased piR-38240 and piR-34393 levels in brains of patients with AD, and these ncRNAs were purported to affect AD pathways (Roy et al., 2017). Furthermore, piR-34393 and piR-38240 both contain potential binding sites for the cytochrome C somatic (CYCS) 3′-UTR. These results confirmed the low expression of CYCS and high expression of the targeting ncRNAs piR-38240 and piR-34393 in individuals with AD. In addition, KPNA6 was reported to be another target of piR-38240. Karyopherin α6, which is encoded by the KPNA6 gene, plays an important role in maintaining cellular homeostasis during oxidative stress (Sun et al., 2011). A low level of KPNA6 expression was correlated with a high level of piR-38240 expression in that study, indicating that piR-38240 contributed to oxidative stress in individuals with AD.

### LncRNA in AD Pathogenesis

#### Roles of lncRNAs in Proliferation and Apoptosis of Neurons

The lncRNA EBF3-AS is a critical lncRNA involved in AD (Magistri et al., 2015). According to Magistri et al. (2015), the expression of the lncRNA EBF3-AS was upregulated in the brains of patients with late-onset Alzheimer’s disease (LOAD). The lncRNA EBF3-AS is transcribed from the opposite strand of the protein-coding gene early B cell factor 3 (EBF3). EBF3, a DNA-binding transcription factor, was reported to inhibit cell proliferation and to lead to cell cycle arrest, growth suppression, and apoptosis (Zhang et al., 2018b). Recently, Gu et al. revealed that EBF3-AS promoted neuronal apoptosis in subjects with AD and was involved in regulating EBF3 expression. Gu et al. observed increased levels of EBF3-AS and EBF3 in the hippocampus of APP/PS1 AD model mice. EBF3-AS knockdown reversed the changes in EBF3 expression and inhibited Aβ-induced apoptosis in SH-SY5Y cells (Gu et al., 2018).

Parenti et al. (2007) observed high expression of NAT-Rad18, the natural antisense transcript of Rad18, in the cerebellum, brainstem and cortex of AD rats. Rad18 is a member of the Rad6 epistasis group that plays an essential role in repairing DNA lesions. Furthermore, Parenti et al. observed the upregulation of NAT-Rad18 and downregulation of Rad18 in rat cortical neurons treated with Aβ_1__–__40_.

In addition, some clinical drugs protect against neuronal injury by regulating specific lncRNAs. For example, simvastatin ameliorates memory deficits and inflammation in clinical and mouse models of AD by suppressing the expression of the lncRNA n336694. Huang et al. revealed marked decreases in the expression of the lncRNA n336694 and miR-106b in brain tissues from APP/PS1 mice treated with simvastatin (Huang W. et al., 2017). The lncRNA n336694 targets the 3′-UTR of miR-106b and increase its expression. In addition, miR-106b induces the expression of p53, Bax, caspase-3 and caspase-9 in SH-SY5Y cells, which were shown to be markers of apoptosis. Simvastatin ameliorates memory deficits and reduces Aβ plaque formation in APP/PS1 mice by suppressing the expression of the lncRNA n336694 and miR-106b (Huang W. et al., 2017).

#### Roles of lncRNAs in Oxidative Stress

The lncRNA SOX21-AS1 plays a key role in oxidative stress-induced neuronal injury in AD mice. As revealed in the study by Zhang et al., the expression of SOX21-AS1, which is the antisense sequence that targets FZD3/5 and regulates the Wnt pathway, is substantially increased in the hippocampal neurons of AD mice (Zhang et al., 2019). Downregulated SOX21-AS1 may potentially increase the expression of FZD3/5 by activating the Wnt signaling pathway, ultimately contributing to the alleviation of hippocampal neuronal oxidative stress, reducing the apoptosis rate and enhancing the learning and memory abilities of mice with AD.

Recently, the lncRNA activation by transforming growth factor-β (lncRNA-ATB) was reported to be involved in AD pathophysiology. Wang et al. discovered increased expression of lncRNA-ATB in the CSF and serum of patients with AD. An Aβ treatment induces PC12 cell cytotoxicity, oxidative stress, and apoptosis and increases the expression of lncRNA-ATB (Wang J. et al., 2018). Suppression of lncRNA-ATB expression alleviates Aβ-induced oxidative stress-induced injury in PC12 cells, possibly by upregulating miR-200.

The lncRNA maternally expressed gene 3 (MEG3) was suggested to be involved in inhibiting oxidative stress and inflammatory injury in subjects with AD. The level of MEG3 is decreased in the tissues of AD rats. MEG3 was identified as a tumor suppressor that inhibits both the p53-dependent and p53-independent pathways (Yi et al., 2019). According to Yi et al., MEG3 expression inhibits the activation of the PI3/Akt signaling pathway in the hippocampal tissues of AD rats. The upregulation of MEG3 contributes to decreased amyloid plaque deposition, the inhibition of oxidative stress and inflammatory injury and the amelioration of the cognitive impairment in AD rats (Yi et al., 2019).

#### The Role of lncRNAs in Regulating Autophagy

The lncRNA 17A regulates autophagy and apoptosis in an AD cell model. Massone et al. (2011) observed increased expression of lncRNA 17A in cerebral tissues derived from patients with AD, and this lncRNA participated in mediating Aβ secretion. Furthermore, Wang et al. (2019) showed that lncRNA 17A regulated autophagy and apoptosis by targeting GABAB receptor 2 (GABABR2), whose expression is significantly increased in SH-SY5Y cells following treatment with Aβ_1__–__42_. GABABR2 has been reported to play a role in regulating microglial function and neuroinflammation (Xu et al., 2018). Upregulated GABABR2 expression inhibits the release of proinflammatory cytokines. The expression of lncRNA 17A decreases the level of the GABABR2 protein; however, silencing lncRNA 17A induces GABABR 2 expression, suppresses cell apoptosis, promotes autophagy, and reduces the ratio of secreted Aβ_1__–__42_/Aβ_1__–__40_ (Wang et al., 2019).

#### The Role of lncRNAs in Regulating the Activity of the Secretases Required for Aβ Production

##### The role of lncRNAs in regulating BACE1

β-site APP cleaving enzyme 1-AS functions as a competing endogenous RNA (ceRNA), sharing miRNA-response elements with BACE1, including miR-29, miR-107, miR-124, miR-485, and miR-761, and positively regulates BACE1 expression in subjects with AD (Faghihi et al., 2008; Modarresi et al., 2011; Zeng et al., 2019). BACE1-AS is expressed at high levels in the blood and brain of patients with AD and in hippocampi from an AD animal model, further facilitating increased BACE1 activity and AD progression. However, BACE1-AS knockdown decreases BACE1 and Aβ levels, inhibits the phosphorylation of the tau protein in the hippocampus and improves the memory and learning performance of SAMP8 mice (Modarresi et al., 2011; Zhang et al., 2018c).

The lncRNA BC200, also known as brain cytoplasmic RNA 1 (BCYRN1), was previously shown to be expressed at high levels in brain tissues from patients with AD (Mus et al., 2007). Recently, Li et al. suggested that BC200 might function as a negative regulator of AD in humans. BC200 facilitates AD pathogenesis by upregulating Aβ production through the modulation of BACE1 expression. The inhibition of BC200 significantly suppresses BACE1 expression, increases cell viability and reduces cell apoptosis in an AD model, and these effects are reversed by BC200 overexpression (Li et al., 2018).

Recently, the lncRNA nuclear enriched abundant transcript 1 (NEAT1) was reported to play an important role in neurodegenerative diseases. NEAT1 was noticeably upregulated and miR-124 was substantially downregulated in an AD mouse model (Zhao et al., 2019). Zhao et al. (2019) identified miR-124 as a direct target of NEAT1 and showed that miR-124 regulates BACE1 expression by binding to its 3′-UTR. Knockdown of NEAT1 or overexpression of miR-124 exerted protective effects on a cellular AD model stimulated with Aβ. Thus, NEAT1 might play a vital role in the development of AD by regulating the miR-124/BACE1 axis.

#### Regulatory Effects of lncRNAs on APP Levels

Sortilin-related receptor 1 (SORL1) is a risk gene for late-onset AD (Xue et al., 2014). A reduction in SORL1 expression promotes an increase in neurotoxic Aβ formation in the brains of individuals with AD, indicating that SORL1 plays an important role in preventing AD progression. SORL1 interacts with APP and functions as a sorting receptor for APP. Downregulation of SORL1 expression shifts APP to the β-secretase cleavage pathway, increasing secreted APPα (sAPPβ) production and promoting Aβ formation (Khvotchev and Sudhof, 2004). Recently, the lncRNA 51A was found to be an antisense configuration of intron 1 in the SORL1 gene that drives the splicing shift of SORL1. The expression of lncRNA 51A is significantly increased in the brains of patients with AD, and it might be involved in Aβ generation in individuals with AD by inhibiting SORL1 expression (Ciarlo et al., 2013; Figure 2).

Neuroblastoma differentiation marker 29 (NDM29) is an RNA polymerase III-transcribed ncRNA, and overexpression of NDM29 might induce APP synthesis and increase β-secretase cleavage, leading to an increase in Aβ secretion that would favor the onset of neurodegenerative processes (Massone et al., 2012; Figure 2). Moreover, Massone et al. also revealed that NDM29 is unequivocally upregulated in the cerebral cortices of patients with AD, possibly contributing to the increase in Aβ secretion.

#### Regulatory Effects of lncRNAs on Aβ Clearance

Low-density lipoprotein receptor-related protein 1 (LRP1) functions in several physiological processes, including the cellular transport of cholesterol, endocytosis of ligands, and transcytosis across the blood-brain barrier (Zlokovic et al., 2010). Recently, LRP1 has been implicated in the clearance of Aβ, and its expression is reduced in individuals with AD (Van Gool et al., 2019; Figure 2). LRP1-AS is a conserved antisense lncRNA transcribed from the LRP1 locus. Yamanaka et al. showed increased expression of LRP1-AS and decreased LRP1 expression in the brains of patients with AD (Yamanaka et al., 2015). Moreover, LRP1-AS negatively regulates LRP1 expression by binding to Hmgb2 and inhibiting the Hmgb2-mediated increase in the transcriptional activity of Srebp1à toward LRP1, indicating that LRP1-AS plays a major role in negatively regulating the systemic clearance of Aβ during AD progression.

### circRNA in AD Pathogenesis

#### Roles of circRNAs in Oxidative Stress

Typically, circRNAs function as miRNA sponges in mammalian cells and have been implicated in the regulation of neuronal diseases, cardiovascular diseases and cancers (Shang et al., 2019). Recently, important regulatory roles were identified for some circRNAs in the oxidative stress pathway, and these circRNAs were potentially useful as therapeutic targets for drugs in the treatment of AD (Figure 2). For example, Huang et al. observed increased expression of mmu_circRNA_013636 (best transcript: *Trpc6*, chr9: 8634046-8,658,377) and decreased expression of mmu_circRNA_012180 (best transcript: *Phkb*, chr8: 88420328-88,446,141) in the hippocampal tissues of SAMP8 mice; however, a Panax Notoginseng Saponin (PNS) treatment reversed the changes in the expression of these circRNAs in the brains of AD model mice (Huang et al., 2018b). Their previous study shown that PNS could prevent oxidative stress injury via increasing the gene expressions and activities of superoxide dismutase (SOD), catalase (CAT), and glutathione peroxidase (GSH-Px) in the brains of SAMP8 (Huang J.L. et al., 2017). Based on these results, the mechanism regulating mmu_circRNA_013636 and mmu_circRNA_012180 expressions may be associated with the therapeutic effect of PNS on AD, and may be closely related to regulator role in oxidative stress.

#### The Role of circRNAs in Regulating Autophagy

Other studies revealed potential roles for circRNAs in autophagosome assembly or vesicular transport-mediated pathways (Figure 2). Huang et al. documented a significant decrease in the expression of mmu_circRNA_017963 (best transcript: *Tbc1d30*, chr10) in 10-month-old SAMP8 mice compared with the controls, indicating that mmu_circRNA_017963 might play an important regulatory role in AD pathogenesis (Huang et al., 2018a). Furthermore, mmu_miR_7033-3p potentially displayed the greatest interaction with mmu_circRNA_017963, which showed a high probability of participating in the biological processes of autophagosome assembly, exocytosis and the synaptic vesicle cycle, functions proven to be associated with AD pathogenesis.

#### The Role of ncRNAs in Regulating the Activity of the Secretases Required for Aβ Production

##### The role of ncRNAs in regulating BACE1

The transcript of the circular RNA sponge for miR-7 (ciRS-7) derived from *CDR1-AS* (antisense to cerebellar degeneration-related protein 1) was previously identified as the endogenous sponge for miR-7, and it plays a crucial role in the pathogenesis of AD. According to previous studies, ciRS-7 is widely expressed in the human brain but is downregulated in the brains of patients with AD. Notably, ciRS-7 alters the expression of ubiquitin protein ligase A (UBE2A), which is essential for the clearance of Aβ in the brains of individuals with AD (Zhao et al., 2016). The downregulation of UBE2A in subjects with sporadic AD is driven by ciRS-7 downregulation and an increase in the expression of endogenous miR-7. UBE2A has been identified as a central effector in the ubiquitin-26S proteasome system that coordinates the clearance of Aβ via proteolysis; it has been shown to be depleted in the brains of individuals with sporadic AD and hence induces amyloid accumulation and the formation of senile plaque deposits. Recently, Shi et al. reported important roles for ciRS-7 in regulating BACE1 and APP protein levels (Shi et al., 2017). The authors found that ciRS-7 did not affect APP or BACE1 gene expression but reduced the levels of the APP and BACE1 proteins. Their follow-up study showed that ciRS-7 overexpression in SH-SY5Y cells increased the levels of the ubiquitin carboxyl-terminal hydrolase L1 (UCHL1) mRNA and protein, which promoted APP and BACE1 ubiquitination and proteasomal degradation. The overexpression of ciRS-7 accelerated the degradation of APP and BACE1 by the proteasomal and lysosomal pathways and reduced the generation of Aβ, indicating a potential neuroprotective role for ciRS-7.

##### The role of ncRNAs in regulating ADAM10

Recently, some circRNAs have been shown to exert important regulatory effects on the apoptotic pathway and the production and degradation of Aβ (Figure 2). For example, the circRNA HDAC9 (circHDAC9) (best transcript: *HDAC9*, chr7:18684293-18,688,306) functions as a miR-138 sponge and is present at significantly lower levels in the serum of both patients with MCI and AD, and its levels are also reduced in mouse and cell models of AD (Lu et al., 2019). Moreover, as shown in the study by Lu et al., the overexpression of circHDAC9 remarkably decreases the miR-138 level and increases the level of the Sirt1 protein to subsequently significantly rescue the miR-138-induced inhibition of ADAM10 and increase in Aβ levels, indicating its potential as a novel early diagnostic marker and therapeutic target for AD.

From the above literature, we found that the dysregulation of ncRNAs caused the certain signal pathways dysfunctions by acting on target genes. These disorders leaded to a series of pathological factors involved in the occurrence and development of AD, such as inflammation, autophagy, oxidative stress and synaptic dysfunction in brain, etc. So, here we summarize the ncRNAs and their downstream target genes. As mentioned in the above literature, these ncRNAs regulated the key genes that might contribute to the physiology and pathology of AD. Whether in cells or animal models, or in human samples, it has been initially shown that these ncRNAs can affect the pathological process of AD. Therefore, by exploring the role of these ncRNAs and the regulating roles in disease pathology, we might found out the potential therapeutic targets for AD.

## The Therapeutic Targets and Diagnostic Biomarkers in AD

### Potential Targets Detected in the Brains of Patients With AD

The expression profile of ncRNAs in the brain tissue of patients with AD during different pathological processes also revealed their potential therapeutic targets and properties (shown in Table 2).

**TABLE 2:** Potential targets detected in the brains of AD cases.

Distribution of biomarkers	Gene	Expression	Source	Pathological stage	Cohort/Country	Samples information	References
**Brain tissue**	miR-455-3p	upregulated	brains tissue of AD cases	AD (Braak stage IV–VI)	AD cases (*n* = 10), MCI cases (*n* = 16), controls (*n* = 14); United States	postmortem brains	Kumar et al., 2017
	miR-501-3p	upregulated	temporal cortex of AD cases	Braak NFT stages IV-VI	AD cases (*n* = 27), controls (*n* = 18); Japan	postmortem brains	Hara et al., 2017
	miR-107	downregulated	temporal cortex of AD cases	MCI (Braak stage III/IV)	AD cases (*n* = 6), MCI cases (*n* = 6), controls (*n* = 14); United States	postmortem brains	Wang et al., 2008; Muller et al., 2014
	miR-132, miR-212	downregulated	temporal cortex of AD cases	Braak stage III/IV (mild AD)	AD cases (*n* = 6), controls (*n* = 6); United States	postmortem brains	Wong et al., 2013; Pichler et al., 2017
	miR-346, miR-153	downregulated	frontal cortex of AD cases	Braak stage VI	AD cases (*n* = 15), controls (*n* = 5); United States	postmortem brains	Long et al., 2012, 2019

Kumar et al. (2017) revealed increased expression of miR-455-3p in the frontal cortex of patients with Braak stages IV-VI AD (representing the progression, usually divided into Braak stages from I to VI), and its expression was significantly upregulated in patients with Braak stage V AD. Wang et al. (2008) revealed reduced miR-107 expression in the temporal cortex of subjects with MCI, and this decrease was sustained in patients with late-stage AD (Braak VI stage). Muller et al. (2014) also observed decreased levels of miR-107 in the hippocampus of patients with early stage AD, although no changes were observed in patients with Braak stage III/IV AD. Based on these results, the level of miR-107 is decreased in human cerebral cortical gray matter early in the pathological progression of AD. Wong et al. (2013) reported a significant reduction in the levels of miR-132 and miR-212 in the temporal cortex of patients with Braak stage III/IV (mild) AD, with slightly more pronounced reductions observed in patients with Braak stage VI (severe) AD. Pichler et al. (2017) also documented the downregulation of miR-132/212 in patients with early stage AD (Braak stage III/IV) with further decreases observed during disease progression (Braak stage > IV). The decreases in the expression of both miRNAs were progressive and substantially more robust in patients with stage V/VI AD than in patients with earlier Braak stages of AD. Long et al. (2019) detected a significantly reduced level of miR-346 in patients with AD, particularly patients in later Braak stages with increased levels of APP. Similar to the results of their previous study, miR-101 and miR-153 were also downregulated in patients with late Braak stage AD, and these changes were accompanied by significantly increased levels of Aβ and APP (Long and Lahiri, 2011b; Long et al., 2012).

The levels of some miRNAs appear to be altered during specific Braak stages and without any differentiation in terms of the degree of disease progression. Changes in the expression of certain miRNAs are observed throughout the entire pathological process, from the middle stage (mild cognitive impairment) to the late phase (Braak VI). Furthermore, these dysregulation of miRNAs in brain of AD cases have also been confirmed to affect the pathological process of AD by regulating target genes and signal pathways, therefore may become the potential therapeutic targets for AD. For example, miR-107 was reported could target BACE1 to inhibit Aβ production in APP/PS1 mouse model. miR-346 specifically targets the 5′-UTR of the APP mRNA to increase APP translation and Aβ production. This information provides insights into whether these miRNAs play a role in driving evolution of the disease.

### Non-coding RNAs as Novel Biomarkers for AD

Early studies focused on identifying differential miRNA expression in the hippocampus of individuals with AD. Recently, researchers have focused on the potential of circulating miRNAs to serve as biomarkers for AD. Several ncRNAs have been identified in the peripheral blood or cerebrospinal fluid of patients with AD and may be useful for clinical applications as diagnostic or prognostic biomarkers of AD (Table 3). Circulating ncRNAs in the serum or CSF are potential candidates for the early detection of AD because their expression changes during the course of disease progression (Kumar and Reddy, 2016). Several recent studies have indicated potential roles for miRNAs as novel biomarkers in the detection and diagnosis of the pathological stage of AD (Dong et al., 2015). Moreover, these miRNA levels correlate with illness hallmarks, including an increased CSF Aβ concentration, hippocampal atrophy and disconnected regions in critical white matter regions of the brain.

**TABLE 3:** Summary of ncRNAs as biomarkers for AD.

Distribution of biomarkers	Gene	Expression	Source	Pathological stage	Cohort/Country	Samples information	References
**Serum**	miR-206, miR-132	upregulated	serum of AD patients	MCI (Braak stage III/IV)	MCI patients (*n* = 66), controls (*n* = 76); China	subjects alive	Xie et al., 2015
	miR-613	upregulated	serum of AD patients	MCI (Braak stage III/IV) and DAT (Braak stage V/VI)	MCI patients (*n* = 32), DAT patients (*n* = 48), controls (*n* = 40); China	subjects alive	Li et al., 2016
	miR-146a, miR-181a	upregulated	serum of AD patients	MCI (Braak stage III/IV)	pMCI patients (*n* = 19), sMC patients (*n* = 26); Italy	subjects alive	Ansari et al., 2019
	miR-455-3p	upregulated	serum of AD patients	MCI and AD	AD patients (*n* = 10), MCI subjects (*n* = 16), controls (*n* = 14); United States	subjects alive	Kumar et al., 2017
	miR-501-3p	downregulated	serum of AD patients	Braak NFT stages IV-VI	AD patients (*n* = 27), controls (*n* = 18); Japan	subjects alive	Hara et al., 2017
	miR-135a, miR-200b	downregulated	serum of AD patients	DAT (Braak stage V/VI)	MCI patients (*n* = 31), DAT patients (*n* = 38), controls (*n* = 30); China	subjects alive	Liu et al., 2014b
	miR-135a, miR-384	upregulated	serum exosomal of AD patients	MCI (Braak stage III/IV) and AD (Braak stage V/VI)	MCI patients (*n* = 101), DAT patients (*n* = 107), controls (*n* = 228); China	subjects alive	Yang et al., 2018
	miR-193b	downregulated	serum exosomal of AD patients	MCI (Braak stage III/IV) and AD (Braak stage V/VI)	MCI patients (*n* = 101), DAT patients (*n* = 107), controls (*n* = 228); China	subjects alive	Yang et al., 2018
**CSF**	miR-613	upregulated	CSF of AD patients	MCI (Braak stage III/IV) and DAT (Braak stage V/VI)	MCI patients (*n* = 32), DAT patients (*n* = 48), controls (*n* = 40); China	subjects alive	Li et al., 2016
	miR-135a, miR-200b	downregulated	CSF of AD patients	DAT (Braak stage V/VI)	DAT patients (*n* = 5), controls (*n* = 5); China	subjects alive	Liu et al., 2014b
	miR-125b, miR-222	upregulated	CSF of AD patients	AD	AD patients (*n* = 10), controls (*n* = 10); Spain	subjects alive	Dangla-Valls et al., 2017
	piR_019949, piR_020364	upregulated	CSF exosomal of AD patients	AD (Braak stage V/VI)	MCI patients (*n* = 17), AD patients (*n* = 42), controls (*n* = 9); Germany	subjects alive	Jain et al., 2019
	piR_019324	downregulated	CSF exosomal of AD patients	AD (Braak stage V/VI)	MCI patients (*n* = 17), AD patients (*n* = 42), controls (*n* = 9); Germany	subjects alive	Jain et al., 2019

Studies have shown an increase in the serum or CSF levels of miR-206, miR-132, miR-613, miR-146a, miR-181a, miR-384, miR-455-3p, miR-125b, and miR-222; however, the levels of miR-501-3p, miR-200b, and miR-193b are downregulated in patients with MCI and AD presenting a progressive cognitive decline, and these alterations are correlated with AD risk factors and markers of pathology. For instance, Xie et al. (2015) found significantly increased serum levels of miR-206 and miR-132 in patients with MCI compared to normal controls. Li et al. observed markedly increased levels of miR-613 in the serum and CSF of patients with MCI and DAT. The change in miR-613 expression was more obvious in patients with DAT than in patients with MCI (Li et al., 2016). Ansari et al. (2019) described significant increases in blood miR-146a and miR-181a levels in patients with MCI who were subsequently diagnosed with AD. Kumar et al. (2017) revealed significantly increased expression of miR-455-3p in the serum of patients with MCI and AD and showed a significant difference in the area under curve (AUC) value. Dangla-Valls et al. (2017) detected the significant upregulation of miR-222 and miR-125b in the CSF of patients with AD. Hara et al. (2017) observed decreased serum miR-501-3p levels and its prominent upregulation in the brain of patients with AD, findings that were significantly correlated with Braak NFT stages, suggesting that changes in the levels of miR-501-3p are related to disease progression. Liu et al. (2014b) documented significantly decreased levels of miR-135a and miR-200b in the serum and CSF of patients with DAT compared with the control groups. Furthermore, the serum miR-200b level in patients with MCI was lower than that in the control group of patients and higher than that in the patients with DAT. Therefore, miR-135a and miR-200b are potential blood-based biomarkers for the diagnosis and staging of AD.

Circulating miRNAs are present in small vesicles known as exosomes within biological fluids, such as human serum. Exosomes isolated from serum have been shown to be highly enriched in miRNAs, and a number of specific miRNAs have been identified as potential biomarkers for AD (Cheng et al., 2015). Yang et al. (2018) noted significantly increased serum exosomal miR-135a and miR-384 levels in patients with MCI and AD compared with patients in the control group. The exosomal miR-193b level in the serum from patients with MCI and AD decreased significantly compared with the serum from healthy subjects.

Significant variability in the changes reported for specific miRNAs has been reported among studies. For example, miR-222 levels are increased in the CSF of patients with AD but decreased in brain tissues from AD mice (Wang et al., 2015). Hara et al. (2017) observed reduced serum miR-501-3p levels and a prominent upregulation of miR-501-3p in the temporal cortex of patients with AD. Serum levels of miR-135a are decreased in patients with AD, yet its levels are increased in serum exosomes.

Various factors contribute to the differences: (1) miRNA stability and degradation, which differs between various classes of miRNA; (2) the use of different methods to quantify miRNA, as the use of microarrays alone is rare and most studies now tend to use quantitative polymerase chain reaction; (3) in certain studies, the rather small cohort of subjects examined is likely a factor associated with problems of data reproducibility. In addition, specific miRNAs are enriched in exosomes but are present at low levels in serum, and they might be diluted in circulating blood, which results in differential expression profiles.

Nevertheless, these miRNAs have great potential as non-invasive and easily detected blood-based biomarkers of AD in patients. These miRNAs may all be involved in the pathogenic mechanisms underlying the progression from MCI to AD and show potential clinical utility as predictive biomarkers.

The use of circulating miRNAs as biomarkers has several advantages, including the close association of these miRNAs with diseases; the non-invasive collection of miRNAs from blood/serum or CSF samples; the easy-to-use technology for miRNA detection; and the stability of circulating miRNAs in serum because of their resistance to RNase digestion, tolerance of different pH conditions, and lack of degradation when stored at room temperature for prolonged periods. These advantages indicate that circulating miRNAs may be ideal biomarkers for AD diagnosis. In the future, studies will be needed to discover more specific miRNAs or other classes of ncRNAs that present the same expression profile in CSF and peripheral blood.

Furthermore, some piRNAs should also be considered as candidate biomarkers for AD. The levels of piRNAs may reflect changes in cellular signaling, which plays important roles in AD pathology. Jain observed increased levels of piR_019949 and piR_020364 and decreased levels of piR_019324 in exosomes derived from the CSF of patients with AD dementia (Jain et al., 2019). The use of these piRNAs as biomarkers was significantly correlated with p-tau levels or the Aβ42/40 ratio, distinguishing patients with AD from controls. In addition, an RNA-sequencing analysis revealed that 146 piRNAs were upregulated and 3 were downregulated in patients with AD compared to healthy controls. Qiu et al. (2017) used an Arraystar HG19 piRNA array to identify 103 piRNAs that were significantly differentially expressed in the brain tissues of patients with AD compared with controls, and these piRNAs were correlated with genome-wide significant risk SNPs. These studies confirmed the important regulatory roles of piRNAs in the pathological processes of AD.

Based on these observations, some ncRNAs exhibit different changes in expression during the pathological processes of AD. These ncRNA profiles are potentially useful as unique biomarkers for the diagnosis of AD, particularly as early diagnostic biomarkers. Early diagnosis may support putative and potential interventions to postpone or prevent subsequent AD onset. Consequently, rapid and non-invasive diagnostic biomarkers must be identified to improve early diagnosis.

## Conclusion

The early diagnosis of AD is difficult to achieve, and timely detection of the disease may offer opportunities for early intervention to potentially delay or suppress the pathological process of AD. Therefore, the identification of reliable and effective biomarkers for the early stages of AD is an effective strategy for AD diagnosis. Circulating ncRNAs that exist in serum, plasma and CSF can be good candidates to be used as diagnostic biomarkers due to their easy identification using simple detection methods and high stability throughout storage and handling. Future studies should focus on the use of highly sensitive RNA analysis methods to confirm the utility of certain ncRNAs as preclinical or clinical diagnostic biomarkers of AD.

In addition, ncRNAs have therapeutic potential, particularly in the preclinical stages of AD. Multiple studies have confirmed that the dysregulation of ncRNAs in animal models plays a critical role in the occurrence and development of AD. Mimicking or inhibitory ncRNAs regulate downstream target mRNAs, thus interfering with the pathological process of AD. Specifically, miRNAs could be considered as the earliest feasible pharmacological targets in AD (Long and Lahiri, 2011a; Junn and Mouradian, 2012). Some studies have confirmed that the use of certain miRNAs as drug targets alleviates or treats AD in murine models of AD (Parsi et al., 2015; Shu et al., 2018). piRNAs are promising molecules in AD therapy. Given the mechanism by which piRNA directly regulate protein expression, synthetic piRNAs might be devised to block the synthesis of AD-related proteins as therapeutic strategies similar to the approaches that have already been tried with miRNAs. LncRNAs and circRNAs represent other interesting classes of candidate molecules for AD therapy. Some lncRNAs and circRNAs with MREs function as miRNA sponges to alter the expression of miRNAs that repress target mRNAs.

Therefore, ncRNAs can be used as potential drug targets in AD due to their regulatory effect on target genes in AD pathology, as evidenced in mouse models and preclinical studies. Table 4 lists parallel changes in miRNA expression observed in mouse models and clinical data.

**TABLE 4:** Changes of ncRNAs expression in murine models and AD patients/cases.

Genes	Changes of ncRNAs levels in murine models of AD	Changes of ncRNAs levels in AD patients/cases
**miRNAs**		
miR-613	serum↑ CSF↑ hippocampus↑, APP/PS1 (3, 6, 9M)	serum↑ CSF↑, MCI/DAT patients
miR-214	hippocampus↓, SAMP8 (8M)	CSF↓, SAD patients
miR-155	cortex↑ hippocampus↑, 3 × Tg AD (12M)	CSF↑, AD patients
miR-16	hippocampus↓, SAMP8 (8, 12M)	frontal cortices↓, AD cases
miR-188	hippocampus↓, 5XFAD (6M)	temporal lobe↓ cerebral cortices↓ hippocampus↓, AD cases
miR-107	hippocampus↓, APP/PS1 (9M)	temporal cortical↓ circulation↓, AD cases
miR-128	cerebral cortex↑, 3 × Tg AD (6, 12M)	monocytes↑ lymphocytes↑, SAD patients
miR-384	hippocampus↓, APP/PS1 (3, 6, 9M)	CSF↓ serum↓, MCI/DAT patients
miR-101	hippocampus↓, cerebral cortex↓, APP/PS1	CSF↓ cerebral cortex↓, AD cases
miR-26b	temporal cortex↑, APP/PS1 (3, 6, 9M)	temporal cortex↑, MCI (Braak III)/AD (Braak VI) cases CSF↑ serum↑, MCI (Braak III)/AD (Braak VI) patients
miR-146a	hippocampus↑, APP/PS1	hippocampus↑, MCI/AD cases CSF↑ serum↑, MCI/AD patients
miR-34c	hippocampus↑, SAMP8 (3, 6, 9M)	serum↑, MCI patients
**lncRNAs**		
EBF3-AS	hippocampus↑, APP/PS1	hippocampus↑ superior frontal gyrus↑ entorhinal cortex↑, LOAD (Braak V-VI) cases
**circRNAs**		
circHDAC9	hippocampus↓, APP/PS1 (4,5M)	serum↓, MCI AD patients

We must discover more ncRNAs and explore new connections in regulatory networks for these ncRNAs to be used as potential diagnostic or therapeutic targets. However, the full potential of ncRNAs as diagnostic or pharmaceutical targets will not be completely realized until we obtain a better understanding of their functions and mechanisms of action in AD.

## Author Contributions

All authors read and approved the final manuscript.

## Conflict of Interest

The authors declare that the research was conducted in the absence of any commercial or financial relationships that could be construed as a potential conflict of interest.

## Abbreviations

3′-UTR: 3′ untranslated region
βAPP: β-amyloid precursor protein
Aβ: beta-amyloid
ABCA1: ATP-binding cassette transporter A1
AD: Alzheimer’s disease
ADAM10: AD-related disintegrin and metalloproteinase 10
AnkG: ankyrin G
AP-1: activator protein-1
apoE: apolipoprotein E
APP: A β precursor protein
ARE: antioxidant response element
BACE1: β-site APP cleaving enzyme 1
BDNF: brain-derived neurotrophic factor
CACNA1C: calcium voltage-gated channel subunit alpha-1 C
CAT: catalase
CDH13: Cadherin 13
ceRNA: competing endogenous RNA
circRNA: circular RNA
CREB-1: cAMP-response-element binding protein
CSF: cerebral spinal fluid
CYCS: Cytochrome C somatic
DAT: dementia of Alzheimer’s type
EGFR: epidermal growth factor receptor
EBF3: early B cell factor 3
GABABR2: GABAB receptor 2
GSH-Px: glutathione peroxidase
HDL: high-density lipoprotein
HEY2: hairy and enhancer of split (Hes)-related with YRPW motif protein 2
IGF-1: insulin-like growth factor 1
IRS1: insulin receptor substrate 1
KPNA6: Karyopherin α 6
LCLs: lymphoblastoid cells
lncRNA: long non-coding RNA
LOAD: late-onset Alzheimer’s disease
LRP1: Low-density lipoprotein receptor-related protein 1
LTP: long-term potentiation
MCI: mild cognitive impairment
miRNA: microRNA
MREs: miRNA reaction elements
ncRNAs: Non-coding RNAs
NDM29: Neuroblastoma Differentiation Marker 29
NFTs: neurofibrillary tangles
NRF2: nuclear factor erythroid 2-related factor 2
Nrp-2: neuropilin-2
piRNA: piwi-interacting RNA
PGC-1α: peroxisome proliferator-activated receptor-gamma coactivator 1α
PMNCs: primary murine neuronal cells
PNS: Panax Notoginseng Saponins
PPARγ: peroxisome proliferator-activated receptor gamma
PTEN: protein phosphatase and tensin homolog
PTGS2: prostaglandin endoperoxide synthase 2
PTPN1: tyrosine protein phosphatase non-receptor type 1
qPCR: quantitative (q)PCR
RISC: RNA-induced silencing complex
ROCK1: rho-associated, coiled-coil containing protein kinase 1
ROS: reactive oxygen species
S1P: ceramide/sphingosine-1-phosphate
SIRT1: silent information regulator factor 2-related enzyme 1, sirtuin1
SNX6: sorting nexin 6
SOD: superoxide dismutase
SORL1: sortilin-related receptor 1
SOX2: sex determining region Y-box 2
SphK1: sphingosine kinase 1
UCHL1: ubiquitin carboxy-terminal hydrolase L1
UBE2A: ubiquitin protein ligase A.
